# A Hybrid Web-Based and In-Person Self-Management Intervention to Prevent Acute to Chronic Pain Transition After Major Lower Extremity Trauma (iPACT-E-Trauma): Protocol for a Pilot Single-Blind Randomized Controlled Trial

**DOI:** 10.2196/resprot.7949

**Published:** 2017-06-26

**Authors:** Mélanie Bérubé, Céline Gélinas, Géraldine Martorella, José Côté, Nancy Feeley, George-Yves Laflamme, Dominique Rouleau, Manon Choinière

**Affiliations:** ^1^ Centre Integré Universitaire du Nord de l'Île de Montréal Departments of Trauma and Nursing Hôpital du Sacré-Cœur de Montréal Montreal, QC Canada; ^2^ Center for Nursing Research Jewish General Hospital McGill University Montreal, QC Canada; ^3^ Tallahassee Memorial HealthCare Center for Research and Evidence Based Practice College of Nursing Florida State University Tallahassee, FL United States; ^4^ Centre de recherche, Centre hospitalier de l’Université de Montréal Faculty of Nursing Université de Montréal Montreal, QC Canada; ^5^ Centre de recherche de l'Hôpital du Sacré-Coeur de Montréal Department of Surgery Universite de Montréal Montreal, QC Canada; ^6^ Centre de recherche, Centre hospitalier de l’Université de Montréal Department of Anesthesia Université de Montréal Montreal, QC Canada

**Keywords:** acute pain, chronic pain, risk factors, protective factors, self-management, early intervention, cognitive therapy, Internet, wound and injuries, lower extremity, feasibility studies, pilot projects

## Abstract

**Background:**

Acute pain frequently transitions to chronic pain after major lower extremity trauma (ET). Several modifiable psychological risk and protective factors have been found to contribute to, or prevent, chronic pain development. Some empirical evidence has shown that interventions, including cognitive and behavioral strategies that promote pain self-management, could prevent chronic pain. However, the efficacy of such interventions has never been demonstrated in ET patients. We have designed a self-management intervention to prevent acute to chronic pain transition after major lower extremity trauma (iPACT-E-Trauma).

**Objective:**

This pilot randomized controlled trial (RCT) aims to evaluate the feasibility and research methods of the intervention, as well as the potential effects of iPACT-E-Trauma, on pain intensity and pain interference with daily activities.

**Methods:**

A 2-arm single-blind pilot RCT will be conducted. Participants will receive the iPACT-E-Trauma intervention (experimental group) or an educational pamphlet (control group) combined with usual care. Data will be collected at baseline, during iPACT-E-Trauma delivery, as well as at 3 and 6 months post-injury. Primary outcomes are pain intensity and pain interference with daily living activities at 6 months post-injury. Secondary outcomes are pain self-efficacy, pain acceptance, pain catastrophizing, pain-related fear, anxiety and depression symptoms, health care service utilization, and return to work.

**Results:**

Fifty-three patients were recruited at the time of manuscript preparation. Comprehensive data analyses will be initiated in July 2017. Study results are expected to be available in 2018.

**Conclusions:**

Chronic pain is an important problem after major lower ET. However, no preventive intervention has yet been successfully proven in these patients. This study will focus on developing a feasible intervention to prevent acute to chronic pain transition in the context of ET. Findings will allow for the refinement of iPACT-E-Trauma and methodological parameters in prevision of a full-scale multi-site RCT.

**Trial Registration:**

International Standard Randomized Controlled Trial Number (ISRCTN): 91987302; http://www.controlled-trials.com/ISRCTN91987302 (Archived by WebCite at http://www.webcitation.org/6rR8G2vMs)

## Introduction

### Acute to Chronic Pain Transition After Major Extremity Trauma

Approximately 65% of traumatic injuries occur in individuals aged 18 to 55 years [1], compromising their most productive years of life. Orthopedic lesions, including lower extremity trauma (ET), affect the majority of injured individuals (80%) [1]. More than 50% of patients with major ET (ie, patients at risk of impaired outcomes usually requiring surgical and multidisciplinary team management) report moderate to severe pain at hospital discharge [2,3], which becomes chronic in up to 86% of cases [4,5]. Pain has several negative consequences. Indeed, poorly managed acute pain has been associated with undesirable outcomes, such as delayed recovery and prolonged length of hospital stay [6,7]. Moreover, intense acute pain has been identified as a chronic pain risk factor after traumatic injury [4,5].

Acute pain is of sudden onset and is expected to last a short time, and can usually be clearly related to a specific event, injury, or illness [8], such as major ET. Chronic pain is defined as ongoing or intermittent, lasting beyond the injury healing process (or more than 3 to 6 months), and adversely impacts daily functioning as well as quality of life (QoL) [9,10]. Healing time after lower ET varies according to the extent of osseous and soft-tissue damage, patient characteristics (eg, age, smoking history, nutritional deficiency), and quality of surgical treatments [11,12], but does not usually exceed 3 months in the absence of complications [11].

People with chronic pain report poorer QoL than individuals affected by common chronic diseases of the heart and lungs [13], making it the most frequent reason for seeking medical attention [14]. Up to 60% of active people who live with chronic pain (including those with lower ET) will experience pain interference with daily living activities, which can lead to job loss or reduced professional responsibilities [15]. Chronic pain also imposes a high socioeconomic burden, which has been estimated to be US $635 billion annually in terms of health care costs and lost productivity [16,17]. Given the impact of chronic pain, the Institute of Medicine (IOM) of the National Academies [8] made “pain prevention” the highest priority for pain relief. To do so, the IOM called for the early promotion of patient self-management behaviors and consideration of biological and psychosocial factors to prevent acute to chronic pain transition.

### Factors Involved in the Development of Chronic Pain

Many longitudinal studies have described chronic pain risk factors. Demographics (eg, female gender, age >65 years, low socioeconomic status) have been shown to play a role in the transition from acute to chronic pain in patients with major ET [4,5]. Moderate to severe acute pain (ie, >4/10 on a numerical rating scale [NRS]) [18] and lower limb trauma are injury-related chronic pain risk factors consistently described in trauma patients [4]. Several potentially modifiable psychological risk factors also seem to be involved, including pain catastrophizing, pain-related fear (ie, kinesiophobia), anxiety, depression, and post-traumatic stress disorder (PTSD) [4,5,19]. However, some patients do not develop chronic pain after ET or other types of injuries, and protective factors have been explored [20,21]. Pain self-efficacy (SE) has been discussed as a protective psychological attribute in acute and chronic pain post-ET [4,22,23]. Pain acceptance has also been associated with improved outcomes in populations that do not include ET patients [24-27].

### Interventions to Prevent Acute to Chronic Pain Transition

Interventions based on a cognitive-behavioral approach have been the most frequently-studied [28] treatments addressing potentially-modifiable psychological factors in the context of chronic pain [29]. These interventions are aimed at promoting individual self-management behaviors (eg, skills to control pain and their effects on physical and psychological functioning) when experiencing pain episodes [30,31]. Such objectives are reached through educational, cognitive (ie, prevention or alteration of maladaptive thoughts, problem-solving) and behavioral (ie, relaxation skills, activity pacing, return to pre-injury activities) strategies as well as complementary approaches, such as support (eg, continued monitoring, encouragement) and relapse prevention (eg, self-monitoring and matching of learned self-management behaviors with real-life situations) [32].

A recent systematic review [29] of psychological therapies for chronic pain management concluded that interventions including such strategies were the most effective. Findings revealed small effect sizes (ie, standardized mean difference [SMD] of 0.15 to 0.29) on pain and related disability and moderate effect sizes (SMD of 0.3 to 0.59) on mood and pain catastrophizing compared to standard care or waiting list. However, these tested interventions have been found to have only small positive effects on pain-related disability and pain catastrophizing compared to active controls (ie, physiotherapy, education, or medical regimens). Most of these effects were observed post-treatment but were not maintained at follow-up (ie, 6 to 12 months after treatment).

Considering the refractoriness of chronic pain to treatment, interest has been growing in the development of interventions intended to promote self-management behaviors before acute pain becomes chronic [28]. Some empirical evidence has revealed that interventions could prevent acute to chronic pain transition several months post-injury, mainly in back pain patients [33-43]. However, no such interventions have been tested in patients with major ET.

In this regard, most randomized controlled trials (RCTs) on preventive self-management interventions have been conducted in patients with pain duration varying from 15 days to 3 months, who were treated in primary care settings or hospital outpatient clinics [33-35,37-43]. The main objective in most of these studies was to restore functioning in activities of daily living and work, and to reduce pain intensity. Most interventions included four to six weekly sessions, which lasted 20 minutes to 2 hours, and were delivered face-to-face individually or in-group. Two to four face-to-face booster sessions to promote the sustainability of intervention effects and/or follow-up phone calls were provided in some studies from two weeks to three months after intervention completion [33,40,42]. Psychologists, nurses, and physiotherapists have most frequently provided these interventions. Research on the effects of such interventions reported positive findings related to pain intensity, restoration of daily functioning, social impact, and psychological variables. Indeed, statistically and clinically significant reductions (between 30% to 50% from baseline) of mean pain intensity scores were observed at 3 and 6 months after the onset of acute pain, favoring the group receiving self-management interventions compared to active controls (ie, education, physiotherapy, biofeedback) [34,41]. Similarly, a study in which participants were classified as back pain “recovered” or “chronic back pain” (based on pain intensity and disability cutoff scores) demonstrated that the number of participants who recovered from their back pain was more than twice as high in the group completing a 4-session self-management program compared to the attention control group (54% vs 23%, *P*=.02) at 6 months [42].

Positive outcomes on work status were also demonstrated in other RCTs documenting that control group participants, receiving usual care or education through written document, were at a 3-to-9-fold greater risk of long-term sick leave (ie, a total of 15-30 or more days in the past 6 months) compared to those receiving the self-management intervention [37-39]. Similarly, patients at risk of chronic back pain showed significant decreases in health care service utilization (ie, self-reported number of visits to physicians or physiotherapists) or were less often referred to multidisciplinary programs for pain at 1-year follow-up when they had received a self-management intervention, in comparison to those who had not.

Furthermore, research findings have shown that the early implementation of self-management interventions designed to increase the patients’ abilities to respond to pain in a more adaptive way contributed to better psychological outcomes. For example, participants who received such interventions demonstrated more positive attitudes towards back pain self-care abilities and significant reductions in pain worry and kinesiophobia at 1-year follow-up [41,43] compared to participants who received education only. Another study showed that participants who received a self-management intervention exhibited less maladaptive behaviors (eg, self-blame) and more adaptive behaviors (eg, problem-focused) [36]. In this regard, experimental group participants were less depressed than the control group subjects, who were 7 times more likely to develop psychopathologies (ie, anxiety, sleep and somatoform disorders) at 1-year follow-up.

Based on knowledge acquired from positive outcomes associated with early self-management programs, we have designed a self-management intervention to prevent acute to chronic pain transition after major lower extremity trauma (iPACT-E-Trauma). Specifically, the intervention focuses on reducing pain intensity and pain interference with daily living activities, to maintain scores <4/10, as recommended by the Initiative on Methods, Measurement, and Pain Assessment in Clinical Trials (IMMPACT) for chronic pain prevention [44]. Patients with an injury to a lower extremity have clinical conditions, recovery profiles, and continuum of care that are different from patients with back pain, necessitating the development of an intervention that fits their specific needs. For example, ET injuries and movement limitations immediately after trauma require the administration of targeted pharmacological and nonpharmacological strategies (ie, cryotherapy, legs being elevated) to reduce inflammation and acute pain in the first intervention sessions, instead of focusing on restoring function. Moreover, ET patients are hospitalized in acute care settings before being transferred to an inpatient and/or outpatient rehabilitation center. The ET patients’ capacity to participate in self-management intervention while in these settings must be considered when selecting the intervention’s dose (ie, duration and number of sessions) and delivery modes. Similarly, the evolving ability of ET patients to bear weight on their injured limb must be considered when guiding them in their return to previous activities, and in the application of pain management strategies (eg, restarting the use of analgesics and cryotherapy for a few days when initially bearing weight on the injured limb).

Empirical and clinical data have guided the development of a preliminary version of the iPACT-E-Trauma intervention, which was refined after the assessment of its acceptability by clinicians (nurses, orthopaedic surgeons, a family physician specialized in pain management, a psychiatrist and physiotherapists) and patients (unpublished observations; Bérubé, Gélinas, Martorella, Feeley, Côté, Laflamme, Rouleau, Choinière; under review). Clinicians and patients positively evaluated the acceptability of the preliminary version of iPACT-E-Trauma. To further improve the suitability (ie, how easy the intervention is applied in the context of daily life) and convenience (ie, willingness to participate in the intervention) [45] of the iPACT-E-Trauma intervention, procedures for the documentation of self-management behaviors by patients were simplified, and session durations were reduced (ie, 15 minutes instead than 30 minutes) as recommended by clinicians. Acceptability assessment of the iPACT-E-Trauma intervention by patients allowed for the tailoring of iPACT-E-Trauma key features based on determinants such as pain intensity, previous knowledge, and the application of self-management behaviors. Furthermore, based on clinicians’ and patients’ input, a Web application was developed to facilitate the intervention delivery in acute care settings. In this paper, an RCT protocol is described to pilot test the refined iPACT-E-Trauma intervention, which is required before evaluating its efficacy in a full-scale multi-site RCT.

### Objectives

#### Primary Objectives

We aim to evaluate intervention and research method feasibility, as well as iPACT-E-Trauma preliminary effects on pain intensity and pain interference (co-primary outcomes) with daily living activities at 6 months post-injury.

#### Secondary Objectives

We aim to: (1) explore the preliminary effects of iPACT-E-Trauma intervention on patients’ perceived pain SE, pain acceptance, pain catastrophizing, pain-related fear, anxiety and depression symptoms, health care service utilization, and return to work (secondary outcomes) at 6 months post-injury; and (2) examine patients’ acceptability assessment of iPACT-E-Trauma [45].

### Hypotheses

We hypothesize that the experimental group will experience a clinically significant reduction of pain intensity (ie, >2 points on a 0-10 NRS) [46] and pain interference with daily living activities (ie, >1 point on a 0-10 NRS) [46] compared to the control group at 6 months post-injury. We expect that the participants in the experimental group will also present higher rates of *no pain* or *mild pain* intensity and/or pain interference scores (ie, <4/10) [44] on these two outcomes. Secondly, we hypothesize that the experimental group will present increased pain SE and pain acceptance as well as reduced pain catastrophizing, pain-related fear, anxiety and depression symptoms, health care service utilization, and increased return to work compared to the control group at 6 months post-injury.

### Trial Design

**Figure 1:**
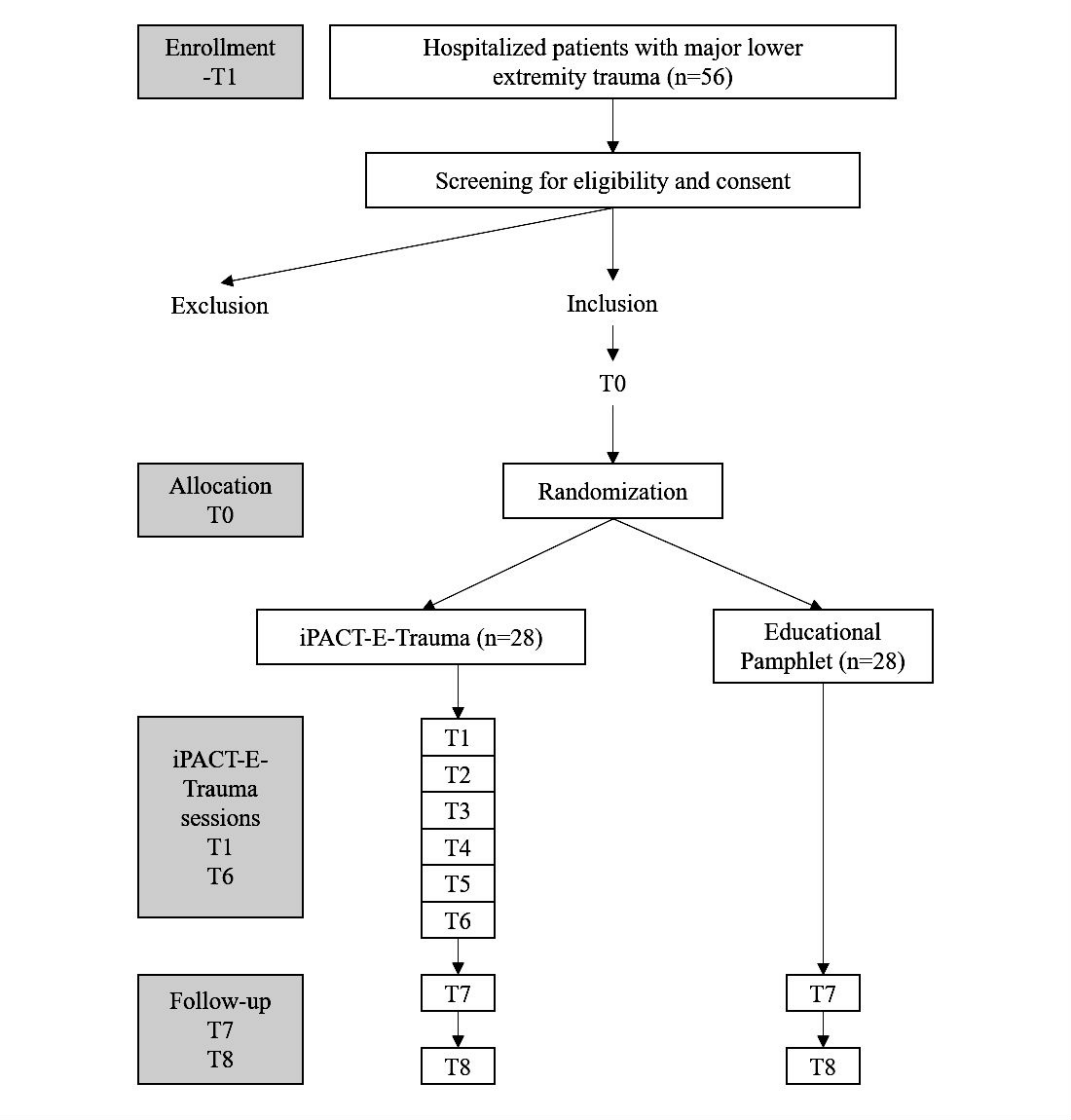
Flow diagram showing the flow of patients in the study protocol.

A 2-arm single-blind pilot RCT will be used. Participants will be randomized to either an experimental group (iPACT-E-Trauma intervention and usual care) or control group (educational pamphlet and usual care) and followed according to the study time points (T1 to T8) presented in Figure 1. The Standard Protocol Items on Recommendations for Interventional Trials (SPIRIT) will be followed [47], per the checklist presented in Multimedia Appendix 1.

## Methods

### Setting

The study will be conducted in a level-1 academic trauma center in Montreal, Canada. The intervention will be provided to the experimental group during their hospitalization in the level-1 trauma center and after hospital discharge. The control group will receive an educational pamphlet while in the trauma center.

### Eligibility Criteria

The inclusion criteria will be: (1) age 18 years or older; (2) able to read and speak French; (3) have a major ET; and (4) at risk of developing chronic pain. Several chronic pain risk factors have been identified in patients with major ET, with acute pain intensity found consistently in this population. IMMPACT has recommended the consideration of this inclusion criterion in chronic pain prevention studies [44]. Patients will be enrolled if they present pain intensity >4/10 upon movement 24 hours post-injury. Waiting 24 hours post-injury to enroll patients will allow their pain intensity to be adequately documented, and will allow us to determine if they are at risk of chronic pain before the administration of the intervention. Considering that lower ET has been identified as a chronic pain risk factor (and to optimize sample homogeneity) only patients with such injuries will be recruited.

The exclusion criteria among ET patients will be: (1) spinal cord injury; (2) other trauma associated with high-intensity pain (eg, >2 rib fractures [48] or surgical abdominal trauma [18]) or principal site of pain not being lower ET; (3) amputation; (4) cognitive impairment (eg, dementia, severe psychiatric disorder, Glasgow Coma Scale score <13/15) [49]; and (5) >7 days of hospitalization. This last criterion was established to minimize delays in intervention delivery, and to standardize intervention timing. Since traumatic injuries occur more frequently in older adults [50], pre-injury somatic pain (eg, pain caused by joint osteoarthritis) will not be an exclusion criterion unless patients report daily analgesic use prior to the trauma. Likewise, patients with a history of visceral pain (eg, inflammatory bowel disease) will not be excluded, considering that this type of pain can be differentiated from musculoskeletal pain caused by a lower ET. Although substance abuse may influence the intervention’s outcomes, this comorbid factor will not be an exclusion criterion. The prevalence of alcohol abuse in patients who have sustained a traumatic injury ranges from 40% to 70% [51-53] and could reach 33% for drug abuse [54]. Excluding patients with a history of substance abuse would impact the external validity of the study. However, data on substance abuse will be collected at baseline and its potential effects on outcomes will be analyzed separately to explain findings, and to inform the research methods of a future full-scale RCT (eg, need for stratified randomization to control for the confounding factor).

### Intervention

#### Control Group

Participants randomized to the control group will receive standard pain management interventions consisting of analgesic administration (ie, opioids and co-analgesia such as acetaminophen and pregabalin) by nurses, according to standardized medical pre-printed orders, and physiotherapy sessions. To ensure that iPACT-E-Trauma effects will not be based exclusively on information and attention patients receive, an educational pamphlet will be distributed to control group participants. The pamphlet will be administered by the research nurse >24 hours (but within 7 days) after hospital admission. The research nurse will visit participants the day after educational pamphlet distribution to answer their questions. Participants will be free to seek medical or other professional care for pain management after hospital discharge.

#### Experimental Group

Participants in the experimental group will receive iPACT-E-Trauma as well as standard pain management interventions. These individuals will be free to seek other pain management treatments after hospital discharge. The biopsychosocial model of chronic pain and empirical data guided the development of the iPACT-E-Trauma intervention; more specifically its ultimate and immediate goals, components, activities, delivery modes, and dose [55].

#### Intervention Content

The biopsychosocial model of chronic pain [56] emphasizes the fact that physical disorders, such as pain, result from dynamic interactions between biological, psychological, and social factors, which may perpetuate and worsen pain. The psychological dimension of the model includes cognitive, affective, and somatic factors, which were taken into account when developing the intervention. *Cognitive factors* refer to promoting pain SE and pain acceptance while minimizing pain catastrophizing and pain-related fear. *Affective factors* relate to emotional reactions, such as anxiety and depression symptoms. It is expected that the intervention’s effects on cognition and affect will influence *somatic factors* by supporting patients in their development of adaptive responses (ie, self-management behaviors) to acute pain, thereby reducing the biologically-related dimension (ie, pain intensity). The development of self-management behaviors and the reduction of pain intensity may then positively influence pain interference with daily living activities, health care service utilization, and return to work, which relate to the social dimension of the biopsychosocial model of chronic pain.

The ultimate goal of iPACT-E-Trauma corresponds to the primary objective of this study, which is to reduce pain intensity and pain interference with daily living activities (co-primary outcomes). Immediate goals relate to determinants that should be changed to manage acute pain and pain interference with daily living activities (ie, to increase pain SE and pain acceptance, and to decrease pain catastrophizing, pain-related fear, and anxiety and depression symptoms [secondary outcomes]). To reach these goals, iPACT-E-Trauma focuses on the following components: (1) the biopsychosocial dimensions of pain and the prevention/regulation of maladaptive thoughts, emotions, and behaviors; (2) the optimal use of pharmacological strategies for acute pain management; (3) the optimal use of nonpharmacological strategies for acute pain management (ie, cryotherapy, leg elevation, relaxation exercises); (4) the adoption of health-promotion strategies (ie, staying active and maintaining an adequate sleep routine); and (5) the return to pre-injury activities. Specific components of iPACT-E-Trauma correspond to several strategies found in cognitive-behavioral interventions (ie, education, problem-solving, activity pacing, graded activity, continued monitoring, encouragement, and matching of learned self-management behaviors with real-life situations). These strategies will be tailored throughout intervention sessions according to the participants’ pain experience, previous knowledge, and adherence to suggested self-management behaviors.

#### Intervention Structure

The iPACT-E-Trauma intervention combines five individual 15-to-30-minute online, face-to-face, and telephone sessions delivered by a nurse starting 24 hours after hospital admission (or after surgery, when required). The intervention also includes two face-to-face or telephone-based booster sessions lasting 15-to-20 in minutes duration. The nurse who will deliver the intervention has received training to deliver the intervention based on a cognitive-behavioral approach (Progressive Goals Attainment [57]). Previous self-management interventions aimed at preventing chronic pain have been delivered individually or to groups, and both strategies have been associated with positive outcomes. Although no such intervention has been provided online or by telephone, these modes were incorporated into iPACT-E-Trauma to facilitate its delivery and to reach patients after hospital discharge. A recent meta-analysis revealed positive results about the delivery of interventions based on a cognitive-behavioral approach in patients with various chronic pain conditions via the Internet alone, or combined with live interactions with clinicians [58]. Telephone-based self-management interventions have shown effects comparable to those in face-to-face interventions in the context of chronic pain [59,60].

According to the intervention dose, most preventive self-management interventions analyzed in the literature review included a total of 4 to 8 weekly sessions of 20 minutes to 2 hours in duration [33,35-39,41,43]. Two to four face-to-face booster sessions were most commonly provided from 2 weeks to 3 months after intervention completion [33,40,42]. A meta-analysis of effective delivery doses in the context of pain supports this time duration [61], since better empirical evidence has been obtained for interventions lasting less than 8 weeks compared to interventions lasting more than 8 weeks. Shorter interventions may improve attendance, give greater learning opportunities, and increase the potential to motivate and engage participants [61].

The iPACT-E-Trauma intervention includes seven short-length sessions to facilitate their integration shortly after injury (Table 1). The first three sessions are planned to be delivered via the Web with the *Traitement et Assistance Virtuelle Infirmière et Enseignement* (TAVIE) platform [62] (ie, *Soulage TAVIE Post-Trauma*) and will be combined with a face-to-face mode of delivery during patient hospitalization. More specifically, participants will be invited to watch a virtual session, followed by a short visit by a research nurse the day after, to answer questions and reinforce learned self-management strategies. Session 2 will be given two days after session 1, and session 3 will be given one week after session 1. The two other regular sessions (ie, sessions 4 and 5) will be delivered on a weekly basis face-to-face during hospitalization or patient appointment in the orthopedic outpatient clinic for medical follow-up, or by telephone if it is not possible to meet the patient in person. Two booster sessions (ie, sessions 6 and 7) will be given by telephone or at the orthopedic outpatient clinic at 6 weeks and 3 months post-injury.

**Table 1:** iPACT-E-Trauma Sessions.

Session	Delivery Timing	Components
1. Web combined with in-person at the hospital	>24 hours to 7 days post hospital admission	Biopsychosocial dimensions of pain: introduction to the biopsychosocial dimensions of pain and how they negatively or positively influence the pain experience
		Self-assessment of pain intensity
		Nonpharmacological pain management/cryotherapy and elevation of legs
2. Web combined with in-person at the hospital	Two days after the first session	Follow-up on previously learned self-management behaviors based on an assessment of patient’s need (ie, pain intensity and adherence to proposed self-management behaviors)
		Pharmacological pain management strategies: analgesics and co-analgesia
		Nonpharmacological pain management strategies/relaxation exercises with a focus on deep breathing relaxation
3. Web combined with in-person at the hospital	One week after the first session	Follow-up on previously learned self-management behaviors based on an assessment of patient’s need
		Biopsychosocial dimensions of pain: prevention/regulation of maladaptive thoughts, emotions and behaviors
		Health promotion/strategies for staying active in the presence of persistent pain: part 1
4. In-person at the hospital or by telephone	One week after the third session	Follow-up on previously learned self-management behaviors based on an assessment of patient’s need
		Health promotion strategies/sleep hygiene
		Health promotion/strategies for staying active in the presence of persistent pain: part 2
5. In-person at the hospital or by telephone	One week after the fourth session	Follow-up on previously learned self-management behaviors based on an assessment of patient’s need
		Pharmacological pain management strategies: how to gradually reduce the consumption of analgesics
		Return to pre-injury activities: establishment of an action plan for returning to pre-injury activities
6. Booster 1: in-person at the hospital or by telephone	Two weeks after the fifth session	Review of the previously learned self-management behaviors if pain intensity >4/10 and/or pain interfering with daily activities on a regular basis
		Pharmacological pain management strategies: how to gradually reduce the consumption of analgesics
		Revision of the plan for returning to pre-injury activities
7. Booster 2: in-person at the hospital or by telephone	Four weeks after the first booster session (3 months post-injury)	Review of the previously learned self-management behaviors if pain intensity >4/10 and/or pain interfering with activities on a regular basis
		Pharmacological pain management strategies: how to gradually reduce the consumption of analgesics
		Referral to appropriate resources if the patient is still experiencing pain intensity >4/10 and taking opioids on a regular basis
		Revision of the plan for returning to pre-injury activities

### Variables and Measurement Tools

A number of variables will be measured at different time points (Multimedia Appendix 2) to meet study objectives based on the SPIRIT statement [47]. Complementary variables will be evaluated to facilitate data interpretation and comparison with other populations.

#### Feasibility

An Intervention Feasibility Evaluation Logbook and Self-Management Behavior Assessment Checklist will allow for the documentation of intervention feasibility data (Multimedia Appendix 3) [55]. The Intervention Feasibility Evaluation Logbook will detail information on intervention components that need to be delivered in each session, the number of webpages and documents consulted on the *Soulage TAVIE Post-Trauma* platform, the appropriateness of the physical environment in which the intervention is delivered, the time dedicated by participants to watch Web sessions, the time required to deliver in-presence sessions by the nurse, and challenges faced in providing the intervention [63]. The Self-Management Behavior Assessment Checklist will allow for the documentation of participants’ engagement and adherence to the intervention (ie, participation in intervention activities and application of recommended self-management behaviors) [63]. A Research Methods Feasibility Form will assess essential criteria of methodological research parameters (Multimedia Appendix 3) [55,64]: (1) adequacy of the sampling pool and recruitment time, (2) ease with which participants are screened, (3) possibility of applying randomization procedures as planned, (4) attrition rate in experimental and control groups, and (5) ease of data collection procedures.

#### Primary and Secondary Outcomes

A comprehensive set of outcome measures will be recorded 24 hours to 7 days post-injury (baseline) and 3 and 6 months later (follow-up). The Brief Pain Inventory (BPI) will measure both primary outcomes (ie, pain intensity and pain interference with daily living activities). Secondary outcomes will be quantified through validated questionnaires: the Pain Self-Efficacy Questionnaire (PSEQ), the Chronic Pain Acceptance Questionnaire-8 items (CPAQ-8), the Pain Catastrophizing Scale (PCS), the Tampa Scale for Kinesiophobia (TSK), and the Hospital Anxiety and Depression Scale (HADS). Health care service utilization will be measured via a Medical Attention Seeking and Professional Services Utilization Logbook, and return to work will be measured with a form that documents work status and responsibility modifications at work. In addition, the Patient Global Impression of Change (PGIC) scale will assess perceived improvement [46]. All instruments have been translated into French using a forward-backward method and/or cultural adaptation. All French version instruments have shown adequate psychometric properties [65-71] as detailed in Multimedia Appendix 4.

##### Brief Pain Inventory

The BPI includes 11 items: 4 on pain intensity (now, average, worst, least) measured on a 0-10 NRS (0=no pain; 10=worst possible pain), and 7 on pain interference with daily living activities, assessed on a 0-10 NRS (0=does not interfere; 10=interferes completely) [72]. The BPI item “walking” was replaced by “mobility (ability to get around)” as proposed in a study performed in persons with cerebral palsy [73] because many patients with major ET may be limited in their walking capacity. Moreover, three additional items (pain interference with self-care, recreational activities, and social activities), proposed in a modified version of the BPI [73], will be added to the Pain Interference with Daily Living Activities Subscale to obtain a broader-based sample of areas that could potentially be affected by pain. Pain intensity upon movement on average in the last 7 days, and total score of pain interference with daily living activities during the same period, will serve as primary outcome measures. Baseline measures of pain intensity and interference will cover the previous 24 hours.

##### Pain Self-Efficacy Questionnaire

PSEQ is a 10-item questionnaire, which assesses the confidence that people with ongoing pain have in their abilities to manage pain and perform activities while in pain [74]. PSEQ scores range from 0 to 49; scores <17 represent very low SE while scores >40 indicate the likely maintenance of behavioral changes, which is the aim of increasing pain SE [74].

##### Chronic Pain Acceptance Questionnaire-8 Items

CPAQ-8 is an 8-item questionnaire measuring pain acceptance. This scale comprises two subscales: the degree to which patients engage in daily living activities regardless of pain (4 items), and the willingness to experience pain (4 items) [75]. No information on relevant score changes has been found.

##### Pain Catastrophizing Scale

PCS comprises 13 items divided into three subscales (rumination, magnification, and helplessness) measuring catastrophizing thoughts [76,77]. Total PCS scores range from 0 to 52; a score of 30 represents a clinically-relevant level of catastrophizing [77].

##### Tampa Scale for Kinesiophobia

TSK is a 17-item checklist developed as a measure of fear of movement/(re)injury in the context of pain [78]. Total score ranges between 17 and 68, with 37 and above differentiating between high and low scores [79].

##### Hospital Anxiety and Depression Scale

HADS is a 14-item inventory divided into two subscales, each comprising 7 items to assess anxiety (HADS-A) and depression (HADS-D) [80]. The range of each subscale is 0-21. Cut-off scores for both subscales indicate that 0-7=normal, 8-10=mild anxiety/depression, 11-14=moderate anxiety/depression, and 15-21=severe anxiety/depression.

##### Patient Global Impression of Change

PGIC [81,82] is a core outcome measure that allows participants to rate their overall improvement regarding their: (1) pain, (2) functioning level, (3) QoL, and (4) global condition [83,84], on a 4-point Likert scale (considerably improved; considerably deteriorated).

#### Acceptability

Two questionnaires will assess intervention acceptability: (1) an E-Health Acceptability Questionnaire that integrates recommended features of Internet-based interventions [85] for Web sessions, and (2) an Intervention Acceptability Questionnaire for in-presence sessions [45]. The E-Health Acceptability Questionnaire has been developed to analyze TAVIE platform content [85], and includes 21 items evaluated on a 5-point descriptive scale, which is divided into 9 subscales: ease of use, ease of understanding, credibility, tailoring, relevance, applicability, visual design appreciation, dosage, motivational appeal, and overall satisfaction with the Internet-based intervention. Experts in the field established content validation of this questionnaire [85].

The Intervention Acceptability Questionnaire is based on a validated instrument intended to describe respondents’ evaluation of intervention acceptability (Treatment Acceptability and Preference Questionnaire: TAP) [45]. The specific acceptability attributes included in this questionnaire are: (1) effectiveness in managing the problem, (2) intervention appropriateness, (3) suitability of the intervention to individual reality, and (4) convenience or willingness to apply and adhere to the intervention. TAP reliability was established in a population receiving behavioral interventions for insomnia (Cronbach alpha >0.80) [45]. Validity was confirmed through factor analysis, confirming 1-factor structure and discriminant validation. TAP has been adapted to evaluate the acceptability of intervention components and activities in this study. Open-ended questions have been added after each item to obtain participants’ input on required modifications to render the intervention more acceptable.

#### Complementary Variables

Short Form 12 items, Version 2

The Short Form 12 items, version 2 (SF-12v2) is a 12-item general measure of health status, facilitating comparisons of outcomes among different disorders and treatments [82]. This scale comprises 8 subscales: physical functioning, role limitation due to physical health problems, bodily pain, general health, vitality, social functioning, emotional health problems, and mental health [86,87]. The scales can be aggregated to provide Physical Component Summary scores and Mental Component Summary scores [88]. SF-12v2 utilizes a linear T-score transformation method so that scores for each of the health domains and component summary measures have a mean of 50 and standard deviation of 10. Scores above and below 50 are above and below the average. Items of the SF-12v2 have been translated into French by a Canadian research team using a forward-backward method, quality ratings of the translated product, and reevaluation of items, as well as pilot testing [89].

##### Injuries and Treatments

An Injury Profile Form will describe injury-related aspects that may affect patients’ pain intensity and recovery. The following aspects will be documented: mechanisms of injury, number of fracture(s), open fracture(s) and their grade, other injuries (eg, mild traumatic brain injury), injury severity score [90], type of treatments received (surgical and nonsurgical), number of surgeries required, soft-tissue injuries, access to compensation, pre-injury chronic pain, substance abuse, technical aids to facilitate ambulation, and fracture healing status.

##### Analgesics Consumption

The Analgesics Consumption Form will describe the analgesics taken by participants during their hospitalization in the trauma center, referring regional hospital, and/or rehabilitation center. After patients are discharged to go home, the number of pills of each analgesic taken will be documented every week. Analgesic consumption will be measured until 6 weeks post-injury, since this timeline has been associated with greater consumption of analgesics after major ET [91].

##### Douleur Neuropathique 4

This 10-item questionnaire documents the presence of neuropathic pain [92], which is a type of pain that is more complex to treat than somatic pain [93]. A score >4 suggests neuropathic pain.

##### Pcl-5

PCL-5 is a 20-item self-report measure that assesses 20 Diagnostic and Statistical Manual of Mental Disorders version 5 (DSM-5) PTSD symptoms [94]. Although iPACT-E-Trauma does not aim to treat PTSD, PCL-5 will be administered, since PTSD has been identified as a risk factor for chronic pain post-ET. PCL-5 has been shown to be reliable (Cronbach alpha=0.94; test-retest reliability r=0.82) and valid (convergent r=0.74-0.85; discriminant r=0.31-0.60; confirmed DSM-5 4-factor model) [94]. Scores range from 0 to 80, and a cut-point of 33 establishes the presence of PTSD [95]. The psychometric properties of the French version have not yet been tested.

### Sample Size

The sample size of a pilot trial should provide reasonable confidence to guide investigators in their decisions about proceeding to a larger trial. Sample sizes that are too large should be avoided due to costs and ethical issues. An 80% 1-sided confidence interval of estimated main trial effect size has been proposed to achieve such aims [96]. Chronic pain prevention studies are not informative about the effect size that should be used to calculate pilot trial sample size. Hence, the effect size documented in chronic pain trials (SMD=0.25-0.35 [29,58]) regarding pain intensity and pain interference with daily activities (primary variables) was used to calculate sample size, and was estimated to be 23 participants per group [96]. With prevision of a 20% attrition rate often encountered in longitudinal studies [64], a total of 56 participants will be recruited and randomized into each group (experimental and control).

### Recruitment

Nurses, medical residents, orthopedic surgeons, and an orthopedic trauma case manager nurse will identify possible participants, inform them about the study, and obtain their permission to be contacted by the principal investigator (PI; MB). These professionals have been informed about the study’s inclusion-exclusion criteria during information sessions. The name and medical file number of patients giving permission to be contacted will be provided to the PI by those who are referring participants via verbal communication or confidential voicemail messages. The PI will then visit these patients. If patients meet the inclusion criteria and wish to participate after receiving a full explanation of the study, the PI will obtain their written informed consent (Multimedia Appendix 5).

### Allocation

#### Randomization

The randomization sequence will be generated by a coordinating center to keep researchers blinded. A computerized random-number generator will produce the sequence. Randomization will be undertaken in permuted blocks of 4 to decrease allocation predictability. Tickets will be placed in sealed, opaque, sequentially numbered envelopes to randomize study participants to either the control or experimental group. Participants will be randomized after obtaining baseline data.

#### Blinding

The research nurse who will administer the intervention cannot be blinded to group assignment considering that she will deliver the iPACT-E-Trauma sessions. To ensure participant blinding to group assignment, the intervention will be delivered (and the educational pamphlet will be distributed) to participants in private hospital rooms, or in hospital rooms in which no other patients are hospitalized for major ET. The research assistant (RA) who will enter data will be blinded to group assignment. A numerical code will be assigned to each participant in the two groups to allow for statistician blinding.

### Data Collection

#### Procedure

At enrollment, the research nurse delivering the intervention will complete the Injury Profile Form and Research Methods Feasibility Form. She will distribute Sociodemographic and Outcome questionnaires to participants. Questionnaires will be placed in sealed envelopes by participants upon completion, to keep the research nurse blinded to the data. An RA will document analgesics given and services received from rehabilitation professionals before patient enrollment.

During intervention delivery, the research nurse will complete the Self-Management Application Checklist to document the rate of self-management behaviors applied in relation to each intervention session. The research nurse will record information concerning intervention delivery and attrition via the Intervention Feasibility Evaluation Logbook and the Research Methods Feasibility Data Form. An RA will fill out the Daily Analgesics Consumption Form while participants are hospitalized. The medical file of participants transferred to referring regional hospitals or rehabilitation centers will be obtained to complete data on analgesic consumption. After discharge to home, an RA will contact patients’ pharmacists to inquire about analgesic distribution. Adequate use of analgesics is a self-management behavior taught within the intervention, so the research nurse will question participants assigned to the experimental group about the remaining quantity of pills in their containers at the beginning of each session, and an RA will phone participants assigned to the control group to question them at corresponding session timelines. A nurse working in the outpatient orthopedic clinic will examine patients for the presence of neuropathic pain by Douleur Neuropathique 4 at each patient’s follow-up appointments. Participants will be invited to fill out the Medical Attention Seeking and Professional Services Utilization Logbook until the conclusion of the study.

Participants in the experimental group will have to complete acceptability questionnaires in the week following sessions 3, 5, and 7. Both groups will also have to complete outcome measures (the SF-12v2 and PCL-5) at 3 and 6 months post-injury. Participants will have the choice of completing questionnaires in hard copy or online (ie, SurveyMonkey). If participants select the hard copy format, acceptability questionnaires will be sent to them by mail and packages of the instruments will be provided to them at medical follow-up appointments in the outpatient orthopedic clinic. Should participants be still hospitalized in a regional hospital or rehabilitation center at the time of completing acceptability questionnaires, an RA will administer them over the phone. Instructions will be provided to participants in the experimental and control groups to return a copy of the Medical Attention Seeking and Professional Services Utilization Logbook at 3 and 6 months. If participants prefer to complete the questionnaires online, an email (including the link for questionnaire access) will be sent to them in the week preceding the 3-month and 6-month data collection points. Phone calls or email reminder(s) by an RA will follow twice, with a 1-week interval if the questionnaires are not completed on time.

#### Data Management

An RA will verify that all questionnaires have been completed at baseline and when they are returned. The RA will hold discussions with participants upon questionnaire completion soon after baseline time point measurement, and contact them by phone at data collection points, to find out why data are missing. If missing data are a result of participant inattention or not understanding a question, the RA will clarify any issues they may have and ask them to provide the missing data. Steps taken to deal with missing data will be noted in the Research Methods Feasibility Data Form.

### Data Analysis

#### Feasibility

To determine the feasibility of iPACT-E-Trauma, rates of planned actions that are applied during interactions with patients, as well as the number of webpages and online documents consulted, will be obtained. Mean time spent by participants watching Web sessions and mean time required for the delivery of intervention sessions by nurses will be computed. Rates of participants’ engagement in intervention sessions and in recommended activities, as well as applied recommended self-management behaviors, will also be calculated. Descriptive data pertaining to intervention delivery challenges will be grouped into categories. Frequencies will be calculated for each category.

Regarding Research Methods Feasibility, descriptive data will be obtained to document: (1) the number of eligible patients and number of participants included; (2) the frequency of recruitment approaches; (3) the mean time required to screen participants relative to their recruitment, consent, and baseline data collection; (4) the percentage of patients who accept to be randomized to either the experimental or control group; (5) the dropout rate relative to each intervention session and outcome measure time points; and (6) the attrition rate at study end [55]. Descriptive data from notes written about difficulties experienced during screening procedures, and obtaining consent and baseline data from patients, will be analyzed by regrouping main difficulties into categories. Frequencies will be calculated for each category. The frequency of unmet inclusion and exclusion criteria will be calculated. If >50% of ET patients are deemed ineligible, the reasons for ineligibility will be analyzed and inclusion/exclusion criteria will be reviewed during the present study, and for the full-scale RCT [55]. Frequency counts on the number of questionnaires not completed in time will be obtained. Mean time between expected dates for questionnaire completion and actual completion will be calculated. Recall rates for questionnaire completion will also be computed.

#### Preliminary Efficacy of the Intervention

All outcome data will be analyzed via an intent-to-treat approach (ie, analyses including all patients randomized, even if they drop out or otherwise have missing data). To estimate differences in primary and secondary variables between the intervention and control groups, a 2-group mixed linear model design with repeated measures on 1 factor (time) will be applied. This model will allow for the detection of significant group differences in continuous outcomes over time due to the intervention. Moreover, the model incorporates the full-information maximum likelihood (FIML) procedure for handling missing data commonly occurring in repeated-measures designs. FIML allows parameter estimation and measures standard errors of parameter estimates, which are statistically nonbiased processes [97,98]. Differences between the experimental and control groups will be analyzed at baseline, 3 months (ie, at the end of intervention), and at 6 months. The results obtained at 6 months will estimate sample size in prevision of a full-scale multi-site RCT.

#### Acceptability

Descriptive analyses of acceptability questionnaire data will be conducted. Frequencies will be calculated for each acceptability attribute. Answers to open-ended questions about required modifications to render the intervention more acceptable in terms of effectiveness, appropriateness, suitability and convenience will be grouped into categories for each session.

### Ethical Considerations

Procedures have been implemented to ensure that the information participants provide in this study will be kept confidential. All participants will be assigned a unique code number. Consent forms will be stored separately from the data. A master list matching the names of participants with their study identification numbers will be kept in a locked filing cabinet separate from the data. No names or other identifying information will appear in any data that is generated.

Study findings will be presented in comprehensive form and not linked to specific participants. All hard copies of the data will be stored in a locked filing cabinet in a locked office. Online survey software secured with enhanced login and encryption features will safeguard the electronic questionnaire format. Data from electronic questionnaires will be downloaded onto a password-protected computer hard-drive and onto a safety code-protected Universal Serial Bus key. Data will be deleted from online survey software at the conclusion of the study, but will be stored for 10 years and then destroyed, and treated as confidential waste.

Protocol amendments will be communicated to the Institutional Review Board (IRB) and through additional information in the trial registration form. Finally, the intervention is not known to be associated with any adverse events. However, such events will be documented if they emerge throughout the study.

## Results

Fifty-three patients were recruited at the time of manuscript preparation. Comprehensive data analyses will be initiated in July 2017. Study results are expected to be available in 2018.

## Discussion

### Study Contributions

The aims of this pilot RCT are to evaluate the feasibility and research methods of a chronic pain preventive intervention. Testing the intervention will provide information on its preliminary efficacy while allowing more data to be gathered on its acceptability from the perspective of patients. Chronic pain has repeatedly been identified as an important health problem in patients with major ET, but no intervention has yet been proposed for them. Evidence from such interventions in other populations is scarce. Indeed, a great deal of attention has been devoted to the implementation of interventions when pain has already become chronic, but few studies have focused on its prevention. This RCT could allow for the development of an evidence-based intervention tailored to ET patients’ needs early after injury.

Preliminary testing of the intervention will set the stage for a full RCT, should the results be promising. If a full RCT shows that the intervention is effective, it will provide professionals of multidisciplinary teams working in the trauma-orthopedics field with scientific guidance and a meaningful way in which to address acute to chronic pain transition. The development of self-management behaviors that decrease long-term reliance on the health care system corresponds to current trends put forward by health policies and health organization decision-makers (ie, to empower patients to take responsibility for their own health and make services more efficient) [8]. A feasible and acceptable intervention aimed at preventing chronic pain could ultimately achieve such objectives while helping to reduce the number of patients with major ET disability and associated social costs.

### Study Limitations

Participation burden is an important limitation of this study. Participants in both groups will be asked to complete numerous questionnaires over a long period of time and participants in the experimental group will also have to attend several intervention sessions. To minimize the risk of attrition related to participation burden, patients will be contacted several times throughout the study by phone or email to complete or remind them to complete questionnaires, which will help maximize continued participation. Participants will receive monetary compensation proportional to the number of outcome questionnaire packages completed and intervention sessions attended (ie, Can $10 per questionnaire package and intervention session). The proposed initial number of participants to be recruited accounts for attrition rates commonly reported in longitudinal studies.

A second limitation pertains to the potential generation of social desirability bias since participants will self-report their performance of intervention activities and self-management behaviors. To minimize this type of bias, participants will be informed that honest answers to questions are crucial for developing interventions that will correspond to ET patients’ needs, and determine if such interventions should be offered to patients with major ET in the future.

### Trial Status

The trial was ongoing at the time of the protocol submission. No comprehensive data analyses had begun at the time of this manuscript preparation.

### Ethical Approval and Consent to Participate

IRB approval was obtained from the *Centre Intégré Universitaire de Santé et de Services Sociaux du Nord-de-l’Île-de Montréal -* *Hôpital du Sacré-Cœur de Montréal (HSCM)* in June 2016 (HSCM Project No. 2017-1333; Multimedia Appendix 6). The PI will obtain written informed consent from participants before initiating the study.

## Appendix

Multimedia Appendix 1 SPIRIT Checklist.

Multimedia Appendix 2 Schedule of enrollment, intervention, and assessments.

Multimedia Appendix 3 Assessment variables for intervention and research methods feasibility.

Multimedia Appendix 4 Psychometric properties of the French version of measurement instruments.

Multimedia Appendix 5 Information and consent form.

Multimedia Appendix 6 IRB approval (original document in French).

## Abbreviations

BPI: Brief Pain Inventory
CPAQ-8: Chronic Pain Acceptance Questionnaire-8 items
DSM-5: Diagnostic and Statistical Manual of Mental Disorders version 5
ET: extremity trauma
FIML: full-information maximum likelihood
FRQ-S: Fonds de recherche du Québec-Santé
HADS: Hospital Anxiety and Depression Scale
HSCM: Hôpital du Sacré-Cœur de Montréal
IMMPACT: Initiative on Methods, Measurement, and Pain Assessment in Clinical Trials
IOM: Institute of Medicine
iPACT-E-Trauma: intervention to prevent acute to chronic pain transition after major lower extremity trauma
IRB: Institutional Review Board
NRS: Numerical Rating Scale
PCS: Pain Catastrophizing Scale
PGIC: Patient Global Impression of Change
PI: principal investigator
PSEQ: Pain Self-Efficacy Questionnaire
PTSD: post-traumatic stress disorder
QoL: quality of life
RA: research assistant
RCT: randomized controlled trial
SE: self-efficacy
SF-12v2: Short Form 12 items, version 2
SMD: standardized mean difference
SPIRIT: Standard Protocol Items: Recommendations for Interventional Trials
TAP: Treatment Acceptability and Preference Questionnaire
TAVIE: Traitement et Assistance Virtuelle Infirmière et Enseignement
TSK: Tampa Scale for Kinesiophobia
