# Supplementary material for: A Hybrid Web-Based and In-Person Self-Management Intervention to Prevent Acute to Chronic Pain Transition After Major Lower Extremity Trauma (iPACT-E-Trauma): Protocol for a Pilot Single-Blind Randomized Controlled Trial
Source: JMIR Res Protoc. 2017 Jun 26;6(6):e125. doi: 10.2196/resprot.7949 (PMC5504342; doi:10.2196/resprot.7949)
Supplement: Multimedia Appendix 2 [file resprot_v6i6e125_app2.pdf]

Schedule of enrollment, intervention and assessments

	Study time points									
	Enrolment	Allocation	Post-allocation*							Closeout
Participants timeline	$-t_1$<br>≥ 24 hours to 7 days post admission	0	t_1<br>S1	t_2<br>S2	t_3<br>S3	t_4<br>S4	t_5<br>S5	t_6<br>S6	t_7<br>S7<br>3-months post-injury	t_8<br>6- months post-injury
Enrolment:<br>Eligibility screen/Informed consent	√									
Allocation of participants		√								
Intervention encounters:										
Control group			√							
Intervention group			√	√	√	√	√	√	√	
Assessments:										
Sociodemographic questionnaire	√									
1. Primary objectives:										
1.1 – Intervention feasibility			√	√	√	√	√	√	√	
1.2 – Research methods feasibility	√	√	√	√	√	√	√	√	√	√
1.3 – Preliminary effects on primary outcomes (BPI questionnaire)	√								√	√
2. Secondary objectives:										
2.1 – Preliminary effects on secondary outcomes (PSEQ, CPAQ-8, PCS, TSK, HADS, PGIC questionnaires, Medical Attention Seeking and Professional Services Utilization Form, Return to Work Questionnaire)	√								√	√
2.2 – Intervention acceptability										
- E-Health Acceptability Questionnaire					√					
- Intervention Acceptability Questionnaire							√		√	
Complementary variables										
- Quality of life (SF-12v2)	√								√	√
- Injuries and treatments (Injury profile form)	√					√				√
- Analgesics consumption (Analgesics Consumption Form)	√		√	√	√	√	√	√		
- Neuropathic Pain (DN4 questionnaire)						√				
- PTDS (PCL-5 questionnaire)									√	√
