# Supplementary material for: A Hybrid Web-Based and In-Person Self-Management Intervention to Prevent Acute to Chronic Pain Transition After Major Lower Extremity Trauma (iPACT-E-Trauma): Protocol for a Pilot Single-Blind Randomized Controlled Trial
Source: JMIR Res Protoc. 2017 Jun 26;6(6):e125. doi: 10.2196/resprot.7949 (PMC5504342; doi:10.2196/resprot.7949)
Supplement: Multimedia Appendix 3 [file resprot_v6i6e125_app3.pdf]

Assessment variables for intervention and research methods feasibility

Variables	Indicators
Intervention feasibility<br>1- The possibility to deliver the intervention as planned	- Consultation of ≥ 80% of web session pages and documents by participants [63].<br>- Provision of ≥ 80% of planned components of each in-presence session and the overall intervention [63].<br>- Sessions duration (sessions 1 to 5: 15 to 30 minutes; session 6 and 7: 15 to 20 minutes).<br>- Challenges faced during intervention sessions delivery.
2- The possibility of participants adhering to the intervention	- Attendance to ≥ 80% of intervention sessions by participants [63].<br>- Engagement in intervention activities and application of recommended self-management ≥ 80% behaviors by participants [63].
Research methods feasibility<br>1- Adequacy of the sampling pool and recruitment time	- Obtaining consent from ≥ 80% of patients approached to participate in the study.<br>- Percentage of eligible patients who were included in the study.<br>- Patient's reasons for refusal to participate in the study.<br>- Difficulties in obtaining patients' consent.<br>- Recruiting study sample (i.e., 56 participants) in approximately 7 months.
2- Ease with which participants are screened	- The time required to screen participants relative to recruitment.<br>- The time required to obtain consent and baseline data relative to recruitment.<br>- Eligibility criteria not limiting the pool of participants by ≥ 50% [55].<br>- Reasons for ineligibility.
3- The possibility of applying randomization procedures as planned	- Difficulties in applying randomization procedures.<br>- Acceptance of patients to be randomized to either the treatment or control group ≥ 80% of the time [55].
4- Attrition rate in experimental and control groups	- Attrition rate in experimental and control groups, i.e., ≤ 20% [64].
5- Ease of data collection procedures	- Rates of questionnaires completed in full.<br>- Number of non-answered questions at each time measure.<br>- Mean time required to complete the outcomes and acceptability questionnaires.<br>- Mean time period between expected dates for questionnaires completion and actual completion.<br>- Recall rates (telephone calls or emails) for questionnaires completion.
