# Supplementary material for: A Hybrid Web-Based and In-Person Self-Management Intervention to Prevent Acute to Chronic Pain Transition After Major Lower Extremity Trauma (iPACT-E-Trauma): Protocol for a Pilot Single-Blind Randomized Controlled Trial
Source: JMIR Res Protoc. 2017 Jun 26;6(6):e125. doi: 10.2196/resprot.7949 (PMC5504342; doi:10.2196/resprot.7949)
Supplement: Multimedia Appendix 4 [file resprot_v6i6e125_app4.pdf]

Psychometric properties of the French version of measurement instruments

	Reliability	Validity
BPI	Cronbach's alphas > 0.8 in cancer patients [65] and Canadian veterans suffering from chronic pain [66].	A two-factor structure and moderately to strongly correlated (r ≥ 0.5) with health status and quality of life in the physical domain [66].
PSEQ	Cronbach's alpha of 0.92 in a chronic pain population [67].	Factor analysis confirmed one factor loading and moderately correlated (r > 0.24 to 0.57) with adaptive coping strategies [67].
CPAQ-8	Cronbach's alpha of 0.75 and test-retest stability at 3 months: r = 0.60, in a population with chronic musculoskeletal pain (personal communication; Scott, Bernier, Garland, Carriere, Sullivan).	Factor analysis confirmed the two CPAQ-8 subscales and moderately correlated (0.28 to 0.5) with depression and disability (personal communication; Scott, Bernier, Garland, Carriere, Sullivan).
PCS	Cronbach's alpha of 0.91 for total score, 0.87 for rumination, 0.57 for magnification and 0.87 for helplessness in undergraduate students during hand immersion in ice water. Test-retest stability at 2 weeks: r ≥ 0.70 [68].	Moderately correlated (r = 0.22 to 0.48) with anxiety, depression, pain intensity and pain tolerance [68].
TSK	Cronbach's alpha of 0.71 in individuals with work-related injuries [69].	Moderately correlated (r = 0.34 to 0.75) with fear avoidance, disability and anxiety [69].
HADS	Cronbach's alphas > 0.8 for the total score and the two subscales in HIV-infected patients [70] and in those consulting for anxiety and depression [71]. Test-retest stability at 6 months: r = 0.72 and 0.74 for total score and the two subscales [71].	Two-factors loading demonstrated [70, 71] and highly correlated (r > 0.67) with other instruments measuring anxiety and depression [75].
PGIC	This instrument measures independent items and responses to treatment at specific time points. No data on reliability were found.	Sensitivity of 79.2% and specificity of 80.1% for the "Very much improved" score and sensitivity of 77.0% and specificity of 78.6% for the "Much improved" score, which correspond to 2-points and 30% improvement on the NRS [83]. Highly correlated (r = 0.42 to 0.78) with the NRS, disability, quality of life and clinician's global impression of change evaluation [84].
SF-12v2	Cronbach's alpha 0.8-0.94 for the 8 subscales [89].	Construct validity: median correlations of items to their hypothesized scales ranged from 0.62 to 0.88) [89].
DN4	Cohen Kappa coefficient 0.66-0.96 [92].	Sensitivity of 82.9% and specificity of 89.9% for a cut-off of 4 [92].
