# Supplementary material for: A Hybrid Web-Based and In-Person Self-Management Intervention to Prevent Acute to Chronic Pain Transition After Major Lower Extremity Trauma (iPACT-E-Trauma): Protocol for a Pilot Single-Blind Randomized Controlled Trial
Source: JMIR Res Protoc. 2017 Jun 26;6(6):e125. doi: 10.2196/resprot.7949 (PMC5504342; doi:10.2196/resprot.7949)
Supplement: Multimedia Appendix 5 [file resprot_v6i6e125_app5.pdf]

Centre intégré
universitaire de santé
et de services sociaux
du Nord-de-
l'Île-de-Montréal
Québec

Information and consent form

Title of the study

Development and preliminary assessment of an intervention aimed at preventing acute to chronic pain in extremity trauma patients - pilot project

Phase 2 – Preliminary assessment of the intervention

Researchers in charge of the study

Student researcher:	Mélanie Bérubé, inf., M.Sc.<br>Doctoral candidate<br>Ingram School of Nursing<br>McGill University
Supervisor:	Dr Céline Gélinas, Ph. D.<br>Assistant professor<br>Ingram School of Nursing<br>McGill University
Members of the thesis committe:	Dr Manon Choinière, Ph. D.<br>Professor<br>Anesthesiology department<br>Université de Montréal
	Dr Nancy Feeley, Ph. D.<br>Assistant professor<br>Ingram School of Nursing<br>McGill University
	Dr Josée Côté, Ph. D.<br>Professor<br>Faculty of Nursing<br>Université de Montréal
	Dr Géraldine Martorella, Ph. D.<br>Associate professor<br>College of Nursing<br>Florida State University

Name of the granting agencies

Canadian Institutes of Health Research (CIHR), Fonds de recherche du Québec - Santé (FRQ-S), Quebec Nursing Interventions Research Network, Quebec Pain Research Network.

Preambule

We are inviting you to take part in a research study. However, before you decide and sign the Information and Consent Form, take the time to read, understand and carefully think about the following information.

This Information and Consent Form may contain information or words that you do not understand. You should ask the researcher in charge of the study or members of the study staff to answer your questions and explain any word or information that you do not understand.

Your participation to multiple studies at the same time could be prejudicial. If so, please inform the researcher.

Nature and objectives of the research project

Patients who have suffered from orthopaedic trauma to lower limbs sometimes develop chronic pain. Several factors (e.g., acute pain intensity, anxiety, ability to self-manage pain) which contribute to the development of chronic pain or protect people from this problem have been identified. We believe that an educational and supportive intervention that takes into account these factors could reduce the pain that people like you sometimes experience long-term, and the difficulties that stem from this in everyday life. We also believe that this type of intervention could contribute to your psychological well-being.

The goal of this research project is to assess whether it is possible to apply the planned intervention, if you can easily adhere to it, and if the methods used to collect data pertinent to this study are adequate. Moreover, this study will allow the assessment of preliminary results associated to the intervention. This would be in anticipation of a bigger study that would be conducted to demonstrate its efficacy.

Targeted participants for this research project are patients over the age of 18 having suffered an orthopedic trauma to one or both lower limbs, at risk of chronic pain, and hospitalized at Centre intégré universitaire de santé et de services sociaux du Nord-de-l'Île-de Montréal (CIUSSS - NIM) - Hôpital du Sacré-Coeur de Montréal (HSCM). We anticipate 56 people participating in this project.

Study procedures

Should you accept to participate in this study, approximately 24 hours following your admission to the hospital or your operation, a research assistant will meet with you to complete questionnaires for a duration of about 30 minutes in order to familiarize themselves with your level if pain and with the interference of pain with your daily activities as well as your psychological condition before the start of the study.

If you've been assigned to the group that receives the intervention, you will be invited to participate in three 10-15 minute online sessions at Hôpital du Sacré-Coeur de Montréal. We will provide you with a computer and headphones, or you can use your own equipment if you prefer. The student researcher will guide you with navigation during your first session. The student researcher will meet with you for 10-15 minutes on the day following the online sessions to answer your questions and reinforce what you have learned. You will then be invited to participate in 4 more sessions of 15 to 30 minutes in person with the student researcher or over the phone. These sessions, along with the meetings with the nurse after each session online, will be digitally recorded in order to ensure that the content and steps of the intervention are adhered to.

The sessions will aim to offer education on pain and its potential effects on your life in general. We will also provide you with support to help you take your medicine and use non-medicinal strategies that aim to relieve pain in an optimal way (e.g., applying ice, sleep). You will also be offered support to help you manage difficult situations that may be caused by pain, and resume activities that are important to you such as family life, sports, studies and work. The student researcher will ask you questions about the medicines you are taking to relieve your pain. You will be given a participant booklet that summarizes the information you will receive and includes the tools to participate in the suggested activities. We will guide you in adequately using the different sections of this booklet.

In addition to this, in the week following the online sessions, the fifth session (6 weeks after your injury) and seventh session (10 weeks after your injury), a research assistant will contact you to ask you questions in order to obtain your comments to determine if the intervention's components, proposed activities, manner in which the intervention was administered (online, face to face, or over the phone) and length of the intervention are acceptable to you, in order to make any required modifications prior to testing the intervention on a large scale. You will also be able to answer these questionnaires electronically by using a secure Internet link. In this case, you will have to provide your e-mail address to the student researcher.

Finally, three months and six months following your injury or the surgery you underwent, we will ask you to complete paper version questionnaires that will be given to you during your follow-up appointment with your orthopaedic surgeon or sent by mail, or an electronic version by means of a secure internet link. These questionnaires will again aim to assess your level of pain, the interference of pain with your daily activities, and your psychological condition. Answering these questionnaires will take about 45 minutes of your time. 6 months following your injury, you will need to submit a table in which we will ask you to record your medical consultations or consultations with other health professionals.

If you have been assigned to the comparison group, you will be given an informational brochure on strategies to relieve your pain soon after your admission to the hospital. The student researcher will visit you to answer your questions the day after you receive the brochure. You will also be contacted by the student researcher at three different times following your discharge from the hospital and she will ask you questions about your medication intake for pain relief.

You will also be invited to fill out paper questionnaires at 3 and 6 months following your injury that will be given to you during your follow-up appointment with your orthopaedic surgeon or sent by mail or an electronic version by means of a secure Internet link. Should you prefer the electronic questionnaires, you will have to provide your e-mail address to the student researcher. These questionnaires will again aim to assess your level of pain, the interference of pain with your daily activities, and your psychological condition. Answering these questionnaires will take about 30 minutes of your time. 6 months following your injury, you will need to submit a table in which we will ask you to record your medical consultations or consultations with other health professionals. Filling in these questionnaires and tables will allow us to assess the preliminary results of the intervention by comparing your answers to these questionnaires to those of participants who will have received the intervention.

Disadvantages associated with the research study

If you are assigned to receive the intervention, the time to participate the intervention (approximately 4 hours) and to answer questionnaires (approximately 3 hours) may appear, for some participants, as a disadvantage.

If you are assigned to the control group, the time required to answer questionnaires (approximately 2 hours) may appear, for some participants, as a disadvantage.

Avantages

You may or may not benefit from taking part in this research study. However, your participation to this study will allow assessing the feasibility of an intervention that may contribute to prevent chronic pain and its negative consequences on the life of people who will have similar injury than you in the future.

Voluntary participation and the right to withdraw

You may choose whether you would like to take part in this study. If you choose to take part now, you can change your mind later and stop at any time and for any reason. Your future medical care and your relationship with your doctor and or other people involved in your care will not change in any way. Tell the researcher in charge of the study or one of the members of the research team of your decision. He or she will explain the best way for you to stop taking part in this study.

The student researcher in charge of the study, the research ethics committee of the Centre integre

universitaire de sante et des services sociaux du Nord-de-l'Île-de Montréal (CIUSSS) - HSCM or the granting agency may take you off the study without your consent at any time if new information shows that taking part in the study is not right for you.

If you choose to stop taking part or are taken off the study, the information that was already collected from you during the study will be stored as long as needed to ensure your safety as well as that of other participants in the study and for as long as the law demands.

There is a chance that we may learn new information while you take part in the research study. This information may change your decision to continue taking part in the study. You will be told any new information, at once, and it will also be given to you in writing.

Confidentiality

While you take part in this study, the student researcher and team will collect and take down information about you in a research study file. Only information necessary for the research study will be collected. The information in your study file could include your age and your injury profile.

All the information collected about you during the study will remain confidential as the law demands. To protect your privacy, your information will be identified with a numbers. Only the researcher in charge of the study knows the numbers that link them to you.

The study researcher will use the study information collected about you for research purposes, only to reach the study goals as they are explained in this Information and Consent Form. The researcher in charge of the study will keep your study information and recordings for 10 years.

The study information could be printed in medical journals or shared with other people at scientific meetings, but it will be impossible to identify you.

To make sure the study is being done properly, your study file as well as your medical file could be checked by a person authorized by the Research Ethics Board of the CIUSS du Nord-de-l'Île-de Montreal - HSCM Hôpital du Sacré-Coeur de Montréal, or by the institution, by a person authorized by special people or groups. All these individuals and organizations adhere to a policy of strict confidentiality.

Funding of the research study

The student researcher received scholarships from the Canadian Institute of Health Research (CIHR), Fonds de recherche du Quebec-Santé (FRQ-S) and of Quebec Nursing Interventions Research Network for the realization of a doctoral project.

Compensation in case of injury and the right of the research participant

If you suffer any injury due to a study procedure, you will receive all the care and services needed to treat you without any cost to you.

By accepting to take part in this study, you keep your legal rights and you do not free researchers or the institution of any of their civil and professional responsibility toward you.

Compensation

You will receive a compensation of 100$ (10$ per session and 10$ per questionnaires package completed) if you are in the intervention and of 30$ if you are in the control group (10$ per questionnaires package completed) for inconvenience because you took part in this study. If you choose to stop taking part or are removed from it before the study is completed, you will be paid only part of this money depending on the time you took part.

Identification of contact person

If you have questions about the study or if you feel you have a problem related to taking part in the study, you can communicate with the student researcher in charge of the study (Mélanie Bérubé) at the following number (514) 338-2222 ext 7366 or her with her supervisor (Céline Gélinas) at (514) 39806157.

If you want to ask questions to a professional or a researcher not involved in this study, you can communicate with Isabelle Roy (clinical nurse in trauma) at (514) 338-222 ext 2655.

If you have any questions to ask regarding your rights as a participant in this study, or if you have complaints or comments you would like to make, you may contact the CIUSS du Nord-de-l'Île-de-Montréal – HSCM Chief executive officer's office at (514) 338-2222 ext 2259.

Control of the ethical aspects of the research study

The Research Ethics Board of the Hôpital du Sacré-Coeur de Montréal of the CIUSSS du Nord-de-l'Île-de Montreal approved this study and is responsible for following the study and making sure that you are protected. Before any change is made to the Information and Consent Form or to the study, it must first be approved by the Research Ethics Board.

Phase 2 – Preliminary assessment of the intervention

Consent

I have read and reviewed the Information and Consent Form and the study was explained to me. My questions were answered to my satisfaction. I was given the time to think about whether I want to take part in this study.

I agree to take part in this study according to the conditions set in this Information and Consent Form. A dated and signed copy of this Information and Consent Form will be given to me.

____
Name of the participant

____ ______________________
Signature Date (day/month/year)

I have explained to the participant the conditions of taking part in the study as stated in this Information and Consent Form and I answered all her/his questions.

____
Name of the person who obtain the consent

____ ______________________
Signature Date (day/month/year)

Signature and commitment of the researcher in charge of the study

I hereby certify that I have explained to the participant the terms of the present information/consent form, that I have answered the questions that the participant had in that respect and that we have clearly indicated that he remains free to withdraw from the study, without suffering any prejudice

I commit myself, as well as the research team, to respect what was agreed upon in the information/consent form and to give a signed copy of this form to the participant.

Name of the student researcher responsible of the research project

Signature of the student researcher responsible of the research project

Date
Day/month/year

AUTHORIZATION OF PHARMACEUTICAL FILE DEMAND

Place your initials beside the statement of your choice	Declaration related to the authorization of pharmaceutical file demand
	**I accept** that my pharmaceutical file be transmitted for consultation in the context of this research project.
	**I refuse** that my pharmaceutical file be transmitted for consultation in the context of this research project.
