# Supplementary material for: A Hybrid Web-Based and In-Person Self-Management Intervention to Prevent Acute to Chronic Pain Transition After Major Lower Extremity Trauma (iPACT-E-Trauma): Protocol for a Pilot Single-Blind Randomized Controlled Trial
Source: JMIR Res Protoc. 2017 Jun 26;6(6):e125. doi: 10.2196/resprot.7949 (PMC5504342; doi:10.2196/resprot.7949)
Supplement: Multimedia Appendix 6 [file resprot_v6i6e125_app6.pdf]

Centre intégré
universitaire de santé
et de services sociaux
du Nord-de-
l'Île-de-Montréal
Québec

APPROBATION D'UN PROJET DE RECHERCHE

NO DE DOSSIER DU CÉR 2017-1333

TITRE: Évaluation préliminaire d'une intervention visant à prévenir la transition de la douleur aiguë à chronique post-trauma orthopédique aux membres inférieures-projet pilote

- *Demande d'évaluation d'un projet de recherche, en date du 9 mai 2016*
- *Protocole, version non datée*
- *Formulaire d'information et de consentement, évaluation préliminaire de l'intervention, version française, version non datée*
- *Formulaire d'information et de consentement, refus de participer à l'étude, version française, version non datée*
- *Lettre d'octroi RRISIQ, en date du 18 décembre 2015*
- *Lettre RQRD, en date du 5 février 2016*
- *Lettre autorisation départementale, en date du 14 octobre 2015*
- *Lettre d'approbation projet par McGill, en date du 22 février 2016*
- *McGill School of Nursing, evaluation form A*
- *Lettre d'appui directrice programme, en date du 8 mai 2016*
- *Lettre octroi de fonds IRSC*
- *Lettre octroi de fonds FRQS, en date du 22 avril 2014*
- *Questionnaires: questionnaire données socio-démographiques, version française, questionnaire concis sur les douleurs (format réduit), version française, questionnaire d'auto-efficacité, version française, questionnaire sur l'acceptation de la douleur chronique – 8 items, version française, échelle de catastrophisation de la douleur, version française, échelle de kinésiophobie de Tampa, version française, évaluation hospitalière de l'anxiété et de la dépression, version française, impression globale de changement, version française, SF-12, version française, douleur neuropa DN4-1, version française, journal de bord consultations, version française, retour au travail, version française, évaluation du partage d'information en lien avec votre participation à l'intervention, version française, questionnaire acceptabilité TAVIE Trauma-Ortho, version française, Questionnaire d'évaluation de l'acceptabilité semaine 5, version française et questionnaire d'évaluation de l'acceptabilité semaine 12, version française*
- *Formulaire de bilan lésionel et de guérison de la ou les fractures, version française*
- *Documents : stratégies pharmacologiques pour une gestion optimale de la douleur, formulaire de consommation des analgésiques et version corrigée, Student Investigator's Intervention Feasibility Evaluation logbook, Self-Care Assessment Cecklist*
- *Demande de consultation de dossiers d'usagers à des fins de recherche, en date du 11 mai 2016*
- *Lettre d'autorisation de consultation de dossiers d'usagers, en date du 11 mai 2016*

LIEU : Centre intégré universitaire de santé et de services sociaux du Nord-de-l'Ile-de Montréal - Hôpital du Sacré-Cœur de Montréal (HSCM), 5400 boul. Gouin Ouest, Montréal (Québec) H4J 1C5

CHERCHEUR(S) : **Mélanie Bérubé, inf., M. Sc.,** Céline Gélinas, Ph. D., Manon Choinière, Ph. D., Nancy Feely, Ph. D., Josée Côté, Ph. D., Géraldine Martorella, Ph. D.

PROVENANCE DES FONDS : Instituts de recherche en santé du Canada et Fonds de recherche Santé Québec

PROBLÉMATIQUE et OBJECTIF DE L'ÉTUDE : Évaluer la faisabilité de la méthode de recherche et d'une intervention visant à prévenir la douleur chronique chez des patients ayant subi un traumatisme aux extrémités inférieures. Évaluer les retombées potentielles de l'intervention sur l'auto-efficacité en lien avec la douleur, l'acceptation de la douleur, la catastrophisation de la douleur, la peur de la douleur, les symptômes de dépression et d'anxiété, l'utilisation des services de santé, et le retour au travail à 6 mois post-trauma.

TYPE DE RECHERCHE : Pilote, essai clinique contrôle, prolongation, sur dossiers

NOMBRE DE SUJETS RECRUTÉS À L'HSCM : 56

CONSÉQUENCES ÉTHIQUES :

Liberté de participer :	Oui	Consentement éclairé :	Oui
Confidentialité :	Oui	Liberté d'en sortir sans contrainte :	Oui

TITRE: Évaluation préliminaire d'une intervention visant à prévenir la transition de la douleur aiguë à chronique post-trauma orthopédique aux membres inférieures-projet pilote

FORMULAIRE DE CONSENTEMENT : (1) évaluation préliminaire de l'intervention
Requis: Oui Version datée du 6 juin 2016 - approuvée

DATE DE L'APPROBATION DU PROJET : 29 juin 2016

DATE DE L'ÉTUDE PAR LE COMITÉ : **Le 6 juin 2016 (séance plénière)**

MEMBRES DU COMITÉ D'ÉTHIQUE DE LA RECHERCHE ET DE L'ÉVALUATION DES TECHNOLOGIES DE LA SANTÉ

AVIS FAVORABLE :
Dre Chantal Lambert, scientifique non-médecin, présidente
Mme Marie-France Thibaudeau, scientifique non-médecin, vice-présidente
Me Marie Boivin, juriste
Mme Henriette Bourassa, membre non affilié représentant la collectivité
Mme Christine Grou, personne spécialisée en éthique
Mme Isabelle Larouche, scientifique non-médecin
Mme Andrée Leboeuf, membre non affilié représentant la collectivité
Dr Yvan Pelletier, scientifique médecin

Absence motivée :
Dre Jadranka Spahija, scientifique non-médecin
Dr Marcio Stürmer, scientifique médecin

Marie-F. Thibaudeau

Marie-France Thibaudeau

29 juin 2016
Date

N.B. : Le Comité d'éthique de la recherche de l'HSCM poursuit ses activités en accord avec *Les bonnes pratiques cliniques (Santé Canada)* et tous les règlements applicables

- La composition de ce comité d'éthique pour la recherche satisfait aux exigences pertinentes prévues dans le titre 5 de la partie C du *Règlement sur les aliments et drogues;*
- Le comité d'éthique pour la recherche exerce ses activités d'une manière conforme aux Bonnes pratiques cliniques;
- Le comité d'éthique de la recherche a examiné et approuvé le formulaire de consentement éclairé et le protocole pour l'essai qui sera mené par le chercheur qualifié susmentionné au lieu d'essai indiqué. L'approbation et les opinions du présent comité ont été consignées par écrit.

Cette approbation est valable pour une période d'un an seulement. Une demande de renouvellement doit être faite après cette période.
